# Stereotactic ablative radiation for pancreatic cancer on a 1.5 Telsa magnetic resonance-linac system

**DOI:** 10.1016/j.phro.2022.10.003

**Published:** 2022-10-28

**Authors:** Kathryn R. Tringale, Neelam Tyagi, Marsha Reyngold, Paul B. Romesser, Abraham Wu, Eileen M. O'Reilly, Anna M. Varghese, Paola Godoy Scripes, Danny N. Khalil, Wungki Park, Kenneth Yu, Christopher H. Crane

**Affiliations:** a Department of Radiation Oncology, Memorial Sloan Kettering Cancer Center, United States; b Department of Medical Physics, Memorial Sloan Kettering Cancer Center, United States; c Department of Early Drug Development, Memorial Sloan Kettering Cancer Center, United States; d Department of Medicine, Memorial Sloan Kettering Cancer Center, United States

**Keywords:** Pancreas cancer, MR linac, MRI, Ablative radiation therapy

## Abstract

**Purpose:**

Ablative radiation therapy (A-RT) appears to improve outcomes in locally advanced pancreatic cancer (LAPC) yet requires solutions for respiratory and digestive motion. We report outcomes of A-RT for pancreatic cancer using 1.5 T MR-adaptive treatment delivery.

**Methods:**

Between March 2020 and July 2021, we treated 30 patients with pancreatic cancer with 50 Gy in 5 fractions (biologically effective dose [BED10] = 100 Gy10) using a novel compression belt workflow and remote planning on the Unity 1.5 T MR linac system. Cumulative incidence of progression was computed from A-RT initiation with death as a competing risk. Overall (OS) and progression-free survival (PFS) were calculated using Kaplan Meier methods.

**Results:**

Of 30 patients, most (73 %) were locally advanced, 4 (13 %) were metastatic, 2 (7 %) were medically inoperable, and 2 (7 %) were locally recurrent. Most (73 %) received FOLFIRINOX prior to A-RT. Median follow-up times from diagnosis and A-RT were 17.6 (IQR 15.8–23.1) and 11.5 months (IQR 9.7–16.1), respectively. Cumulative incidences at 1-year of local and distant progression were 19.3 % (95 %CI 6.7–36.8 %) and 47.4 % (95 %CI 26.7–65.6 %), respectively. Median OS from diagnosis and A-RT were not reached. One-year OS from diagnosis and A-RT were 96.4 % (95 %CI 77.2–99.5 %) and 80.0 % (95 %CI 57.3–91.4 %), respectively. Median and 1-year PFS were 10.1 months (95 %CI 4.4–14.4) and 39.7 % (95 %CI 20.3–58.5 %), respectively. No grade 3 + toxicities were observed.

**Conclusions:**

A-RT using the 1.5 T Unity MR Linac resulted in promising LC and OS with no severe toxicity in patients with LAPC despite radiosensitive organs adjacent to the target volumes. Longer follow-up is needed to assess long-term outcomes.

## Highlights

• Ablative radiation therapy (A-RT) is effective for locally advanced pancreas cancer.
• 30 patients received A-RT using diagnostic quality MR-adaptive treatment delivery.
• Cumulative incidences of 1-year local and distant progression were 19.3% and 47.4%
• Overall and progression-free survival 1-year from A-RT was 80.0% and 39.7%
• 50 Gray in 5 fractions led to promising 1-year local control and survival.
• Most local failures were marginal at the tumor-organ-at-risk (OAR) interface.
• No grade 3 or higher toxicities were observed despite adjacent sensitive OARs.

## 1 Introduction

Pancreatic cancer incidence is rising and expected to be the second leading cause of cancer-related death by 2040 [1], [2]. Treatment options for locally advanced unresectable pancreatic cancer (LAPC) have significant limitations. The median survival has consistently been reported to be between 10 and 12 months[3]. Improvements in systemic therapies seem to have modestly improved survival outcomes [4]; however, studies often include patients who were able to have surgery and median survival results have improved as the proportion of resected patients increases [5], [6]. Approximately-one-third of patients with LAPC die without distant metastases and many others die from complications related to local disease in the presence of limited distant disease [7], [8]. Effective local control with either surgery or ablative radiotherapy (RT) prevents significant complications related to local progression, such as portal vein, biliary, and duodenal obstructions, leading to marked improvement in survival over other palliative treatments [5], [7].

It has been appreciated widely for decades that respiratory and digestive motion create uncertainty in the treatment of upper abdominal malignancies. Earlier RT approaches, including low dose stereotactic body radiotherapy (SBRT), were unable to address digestive motion and thus restricted radiation dose to palliative levels (i.e., 54 Gray [Gy] in 30 fractions) for safety [9], [10], [11], [12], [13]. RT for unresectable disease using palliative doses failed to improve survival in the most definitive study, the LAP-07 trial [14], thus demonstrating the need for more effective local approaches. Sparing the mobile luminal organs at risk (OARs) surrounding the pancreas is critical to safely deliver definitive doses to pancreas tumors. We reported results using ablative doses (i.e., approximately 100 Gy biological-effective dose [BED10]) using hypofractionated regimens over 3–5 weeks that address digestive motion by allowing the motion to volume average over 15 to 25 fractions [15]. The high local tumor control rate seems to have prevented tumor-related morbidity leading to improved long-term survival in much the same way that surgery does [15], [16].

Another way to address digestive motion is to correct for it directly with adaptive planning [17], [18]. MR-guided RT (MRgRT) systems enable delivery of intensity modulated radiation while correcting for respiratory and digestive motion via daily adaptive planning and MR image guidance [17], [18], [19], [20], [21], [22]. These techniques have allowed for dose-escalation to definitive levels, and initial studies have demonstrated improved survival [23], [24], [25]. As such, the MR linac introduces many advantages that may make ablative dose delivery near the mobile GI tract commonplace. As technologies are still evolving, we have developed useful interim respiratory motion and workflow solutions for use on the 1.5 T MR linac, which have enabled the routine treatment of patients with 50 Gy in 5 fractions [26]. The primary aims of this study were to 1) report our current treatment approach and 2) demonstrate encouraging clinical outcomes of 30 patients treated with an ablative dose for LAPC on the MR linac.

## 2 Materials and methods

### 2.1 Patient population

All patients with pancreatic cancer (n = 30) treated between March 2020 and July 2021 on the Elekta Unity 1.5 T MR linac system were included. Resectability was adjudicated via multidisciplinary discussion in a dedicated pancreatic tumor conference. This study was approved by the retrospective Memorial Sloan Kettering IRB protocol #21–129. Informed consent was waived given the retrospective study nature.

### 2.2 RT simulation workflow

The details of our simulation workflow with an abdominal compression belt have been described in our previous publication [26]. Here, we provide an update to our simulation workflow. As motion management using gating and tracking is in development for the Elekta Unity, we use abdominal compression as an interim solution for respiratory motion. Details of simulation set up and beam arrangement are described in Supplementary Fig. 1.

### 2.3 RT planning details

The gross tumor (GTV) target dose was 50 Gy in 5 fractions (BED assuming ⍺/β = 10, BED10 = 100 Gy) along with a second dose level of 25 or 33 Gy in 5 fractions to the volume at risk of harboring microscopic disease. Planning treatment volume procedures exclude sensitive OARs from high dose treatment volumes as previously described [16]. Supplementary Fig. 2 provides details of the approach to target and OAR contours.

## 3 Treatment workflow on Unity

**Fig. 1:**
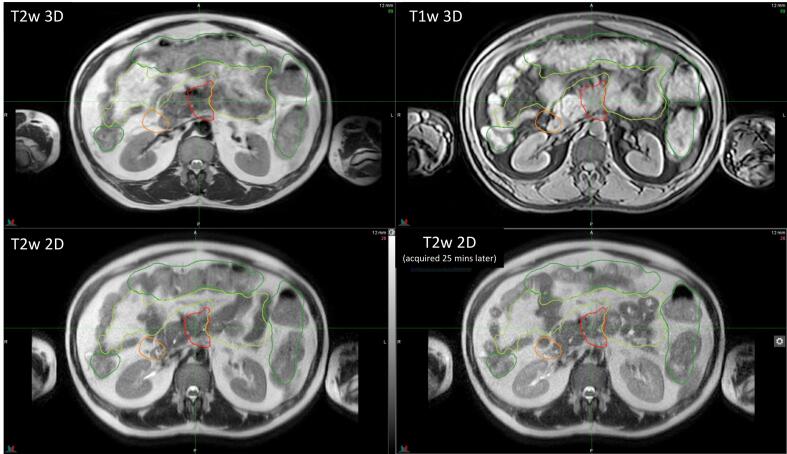
**Multiple MR sequences used for online contouring.** Multiple MR sequences were used to contour targets (GTV in red) and OARs (stomach_duodenum in orange, small bowel in light green, large bowel in dark green). These sequences included T2w 3D (TR/TE = 1300/87, FOV = 400x440x250, slice thickness = 2 mm; top left), eTHRIVE (T1w 3D with fat saturation TR/TE = 4.6/2.3, FOV = 400x450x250, slice thickness = 2 mm; top right) and single shot T2w 2DMRI (TR/TE = 1250/80, FOV = 400x350x200, slice thickness = 4 mm; bottom). (For interpretation of the references to color in this figure legend, the reader is referred to the web version of this article.)

Each patient underwent 5-fraction A-RT treatment with daily online plan adaptation using Elekta’s Adapt-to-Shape (ATS) workflow (Supplementary Fig. 3). The standard ATS workflow, as described in our previous publication [26], was modified to enable contouring in MIM VISTA^TM^ that allowed the use of multiple MR sequences (instead of a single 3D sequence) for contouring (Fig. 1). In addition, fast 2-minute T2w 2D MRIs were also acquired every 5 min and sent to MIM to assess potential intrafraction motion during contouring**.** Patients were given lorazepam and loperamide to minimize discomfort from the belt and slow bowel movements, respectively. Patients were also given half a cup of water to help differentiate duodenum and GTV interface.

## 4 Follow-Up and outcomes assessment

After A-RT, patients were followed with a CT or MRI of the chest, abdomen, and pelvis and blood work at 1-month post-A-RT then every 3 months thereafter. Local, regional, and distant disease progression were characterized by follow-up imaging and/or confirmatory biopsy if performed. Local progression was classified per RECIST 1.1 criteria. Regional failure was defined as regional lymph node recurrence outside the initial PTV50. Patients were also considered to have progression based on clinical or biochemical factors (i.e., worsening symptoms or rising CA19-9). Acute toxicity was evaluated per the National Cancer Institute’s Common Terminology Criteria for Adverse Events (CTCAE) version 5.0 for events occurring within 90 days of the first fraction of A-RT. Late toxicities were those occurring > 90 days from the first fraction of A-RT and were defined by the Radiation Therapy oncology Group/European Organization for Research and Treatment in Cancer Late Radiation Morbidity Scoring Schema [27].

### 4.1 Statistical analysis

The primary endpoint was local tumor control evaluated as the cumulative incidence of local disease progression. The cumulative incidence was computed from the date of first fraction of A-RT, and patients were censored at the date of most recent abdominal imaging with death as a competing risk. Cumulative incidence of distant progression was also calculated. Local progression did not preclude a patient from having a distant disease progression event and vice versa. Local control was also calculated by Kaplan Meier method with patients censored at the date of last follow-up. Overall survival (OS) was measured from both the date of diagnosis and first fraction of A-RT. Progression-free survival (PFS) was measured from the date of first fraction of A-RT through first progression (local, regional, or distant) or death. For both survival endpoints, patients were censored at the date of last follow-up and Kaplan Meier curves were generated.

## 5 Results

Clinical and demographic characteristics for the 30 patients are described in Table 1. The median age was 67 years (range, 57–95) and most patients had high performance status (87 % Karnofsky Performance Status [KPS] > 70). More patients had a tumor located in the pancreatic body or tail (53 %). At time of A-RT, most patients had locally advanced, unresectable disease (73 %), four (13 %) had evidence of metastatic disease, two (7 %) were medically inoperable, and two (7 %) were locally recurrent after prior distal pancreatectomy. Median CA 19–9 was 92 U/mL at initial diagnosis (range, 3–13,198) and 58 U/mL at A-RT initiation (range, 0–2,363), with most patients experiencing a decrease prior to A-RT (70 %). Most patients received induction FOLIRINOX (73 %). Some patients received concurrent checkpoint inhibitor on protocol (30 %).

**Table 1:** Patient and treatment characteristics.

**Characteristic**	**N (%), Median (range)**
Age (years) at A-RT start	69 (57–95)
Gender	
Male	18 (60 %)
Female	12 (40 %)
Clinical Presentation	
Abdominal pain	18 (60 %)
Jaundice	3 (10 %)
Weight loss	16 (53 %)
Diabetes	8 (27 %)
Tumor location	
Head	14 (47 %)
Body/tail	16 (53 %)
KPS at A-RT start	
≤70	4 (13 %)
>70	26 (87 %)
Stage and Resectability	
Locally advanced, unresectable	22 (73 %)
Resectable, medically inoperable	2 (7 %)
Locally recurrent	2 (7 %)
Metastatic	4 (13 %)
Nodal Status	
N+	10 (33 %)
CA 19–9 (U/mL)	
Initial diagnosis	92 (3–13,198)
At RT start	58 (0–2,363)
Decline pre-RT	21 (70 %)
% Decrease	55 (-100 to 100)
At RT end	43.5 (0–2,552)
CEA (U/mL)	
Initial diagnosis	3.5 (0.9–30.7)
At RT start	3.95 (0.8–10.5)
Decline pre-RT	7 (23 %)
At RT end	4.8 (1.7–13)
Induction chemotherapy, N (%)	
FOLFOX	1 (3 %)
FOLFIRINOX*	22 (73 %)
Gemcitabine/nab-paclitaxel	1 (3 %)
Gemcitabine/abraxane	1 (3 %)
None^c^	5 (17 %)
Concurrent Systemic Treatment	
None	21 (70 %)
Checkpoint inhibitor	9 (30 %)
Radiation dose	
Total prescribed dose (Gy), median	50
Total prescribed fractions	5

* Includes mFOLFIROX. One patient treated with A-RT for recurrent pancreatic cancer had prior adjuvant FOLFIRINOX but no chemotherapy pre-A-RT at the time of recurrence.

**Fig. 2:**
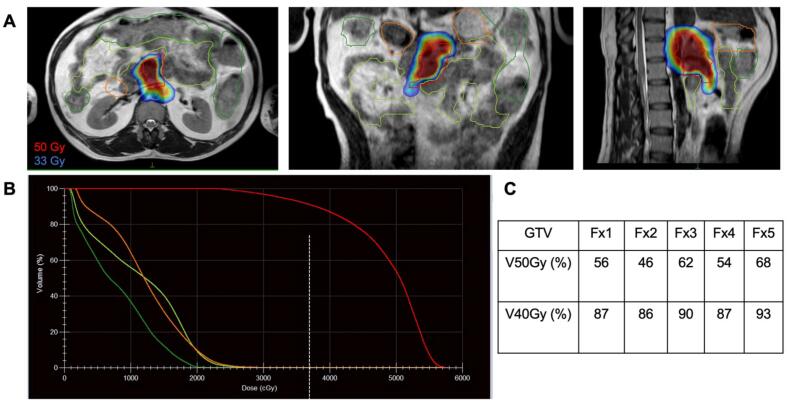
**Dosimetry for an example case.** A) Dose color-wash demonstrates heterogeneity of high dose (50 Gy) coverage of the GTV to prioritize sparing the adjacent OARs (small bowel in green, stomach_duodenum in orange), which allows for treatment of anatomically challenging tumors with radiosensitive structures wrapped around the target. Dose coverage shows lower prescription coverage of the GTV to prioritize sparing the adjacent OARs. B) Dose-volume histogram (DVH) demonstrates dosimetry for an example fraction and C) includes a table summarizing the target coverage across all five fractions. Abbreviations: Fx, fraction; VXXGy, volume receiving XXGy (%). (For interpretation of the references to color in this figure legend, the reader is referred to the web version of this article.)

Daily online plan adaptation was performed to account for day-to-day variation in OAR positioning or to improve target coverage**.** Table 2 shows the pre-treatment as well as adaptive planning dosimetry in terms of target coverage and OAR sparing. Median target coverage and OAR sparing between pre-treatment plans and adaptive plans were comparable. Prescription coverage to GTV during adaptive planning was slightly lower (66.5 % vs 72.7 %) compared to pre-treatment due to large variation in OAR positioning. OAR sparing within our institutional guidelines took priority during planning constraints. Dose distribution using color-wash with a dose volume histogram (DVH) in an anatomically challenging case with radiosensitive OARs surrounding the GTV is shown in Fig. 2.

**Table 2:** Target dosimetry and constraints with pre-treatment and on-treatment dosimetry.

		**Median (Range)**		**Mean±SD**	
	**Target constraints**	**Pre-treatment dosimetry**	**Adaptive Planning**	**Pre-treatment dosimetry**	**Adaptive Planning**
GTV	V40Gy > 90%	93.9	91.8	92.5 ± 7.4	91.0 ± 5.6
		(63.6 – 100)	(75.6 – 100)		
	V50Gy > 90%	72.7	66.5	72.1 ± 15.6	68.0 ± 11.9
		(33.4 – 97.2)	(44.0 – 95.6)		
PTV50	D0.035 cc < 60 Gy	57.3	57.4	57.5 ± 0.9	57.6 ± 1.0
		(56.2 – 59.5)	(56.2 – 60.0)		
	D95% > 50 Gy	39.4	38.1	39.4 ±3.5	38.0 ± 2.9
		(32.9 – 46.0)	(32.1 - 43.8)		
Small Bowel	D0.035cc < 33 Gy	32.3	31.7	31.7 ± 1.5	31.3 ± 1.7
		(26.1 – 33.0)	(24.6 – 33.1)		
	D5cc < 25 Gy	24.8	24.6	24.0 ± 1.4	23.9 ± 1.3
		(20.4 – 25.0)	(20.1 – 25.0)		
	V20Gy < 100 cc	23.6	27.1	28.5 ± 19.4	28.7 ± 16.9
		(5.6 – 83.7)	(5.1 – 70.1)		
Small Bowel PRV	D2cc < 33 Gy	28.6	28.8	28.3 ± 1.9	28.2 ± 1.9
		(24.3 – 33)	(22.1 31.4)		
Stomach_Duodenum	D0.035cc < 33 Gy	32.6	32	32.3 ± 0.8	31.7 ± 0.8
		(29.9 – 33)	(29.2 – 32.8)		
	D5cc < 25Gy	24.9	24.7	24.4 ± 0.9	24.3 ± 8.1
		(20.5 – 25.0)	(21.1 – 25.0)		
Stomach_Duo PRV	D2cc < 3300	29.6	28.9	29.5 ± 1.4	28.8 ± 1.5
		(26.3 – 32.9)	(24.5 – 32.2)		
Large Bowel	D0.035cc < 33 Gy	32.2	29.6	28.8 ± 5.5	27.8 ± 4.2
		(12.5 – 33.1)	(19.4 – 32.7)		
	D5cc < 30 Gy	24	22	22.9 ± 4.2	22.2 ± 3.8
		(9.4 – 27.6)	(14.2 – 27.8)		
Large Bowel PRV	D2cc < 33 Gy	26.9	24.5	26.0 ± 5.2	25.1 ± 4.1
		(10.4 - 32.8)	(17.1 – 31.0)		

Abbreviations: GTV, gross target volume; PTV50, planning target volume prescribed 50 Gy; PRV, planning organ at risk volume; VXXGy, volume receiving XXGy (%); DXXcc, dose to XXcc volume (Gy); SD, standard deviation.

**Fig. 3:**
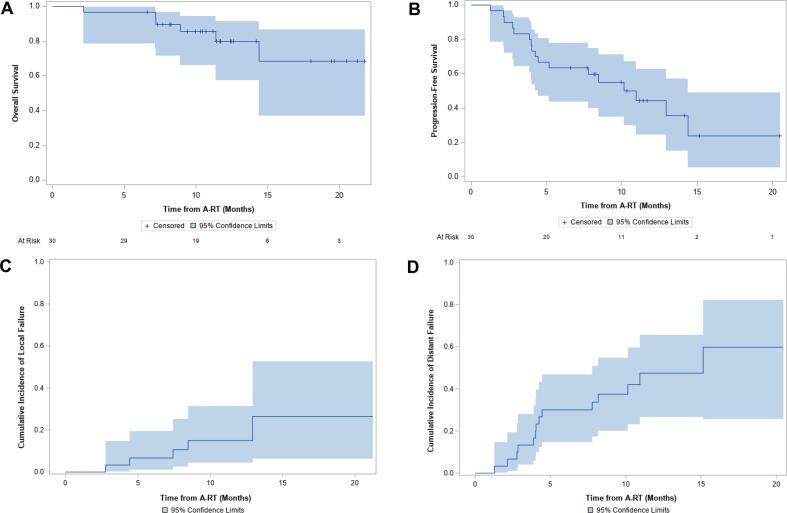
**Overall, progression-free survival, and cumulative incidences of progression.** A) Overall survival (OS) and B) progression-free survival (PFS) measured from date of first fraction of ablative RT. Cumulative incidence with death as a competing risk of (C) local and (D) distant progression from date of first fraction of ablative RT. Abbreviations: A-RT, ablative radiotherapy.

Of the 24 living patients, 14 have evidence of active disease and 10 have no evidence of active disease. Median PFS was 10.1 months (95 %CI 4.4–14.4) with 1-year PFS of 39.7 % (95 %CI 20.3–58.5; Fig. 3**b**). Six patients had local disease progression (Supplementary Fig. 4). Two local disease failures were marginal, occurring at the interface of the GTV and a critical, radiosensitive organ: one at the transverse duodenum, the other at the stomach. One patient was judged to have local progression despite stable mass size given FDG avidity of the GTV and rising CA 19–9 triggering initiation of capecitabine. One local progression was preceded by a distant metastasis (lung) 8 months prior and two local progressions had synchronous distant metastases (both liver). Two patients had regional disease progression involving new and increased gastrohepatic nodes that were not included in the original GTV or PTV50 but had been covered by the low dose (25 Gy) elective region. Local control at 1-year was 78.8 % (95 %CI 55.8–90.7) and the median time to local progression was 8.0 months (range 2.8–12.9). The cumulative incidence of local failure at 6 months was 6.7 % (95 %CI 1.1–19.5 %) and 1-year was 19.3 % (95 %CI 6.7–36.8 %; Fig. 3**c**). The cumulative incidence of distant metastases at 6 months was 30.0 % (95 %CI 14.8–46.9 %) and 1 year was 63.4 % (95 %CI 27.3–85.4 %; Fig. 3**d**).

Median follow-up time among survivors from diagnosis was 17.6 months (interquartile range [IQR], 15.8–23.1) and 11.5 months (IQR, 9.7–16.1) from the first fraction of A-RT. At the time of this analysis, 6 patients had died, and all but 1 patient had evidence of disease at death. One patient died from multiple strokes after thoracentesis, 1 died from sepsis in the setting of recurrent cholangitis, 1 died likely from sepsis after a biliary stent exchange, and 3 died of unclear causes. Median OS from diagnosis was not reached (95 %CI 20.1-not reached) with 1- and 2-year OS of 96.4 % (95 %CI 77.2–99.5 %) and 70.8 % (95 % CI 44.2–86.4 %), respectively. Median OS from the first fraction of A-RT was also not reached (95 %CI 14.4-not reached; Fig. 3**a**). OS at one year from A-RT was 80.0 % (95 %CI 57.3–91.4 %).

All patients were able to complete the entire course of A-RT treatment. Toxicities are described in Table 3. Acute grade 2 toxicities occurred in 4 patients (13.3 %). There were no grade 3 or higher acute toxicities related to A-RT. One patient did experience a grade 3 variceal bleed, the etiology of which was judged by the treating physician to be venous congestion in the setting of superior mesenteric vein thrombosis. This patient also had a history of grade 3 variceal bleeds, most recently just one month prior to A-RT, and had previously been on anticoagulation (held prior to A-RT). A-RT was held following three fractions for a patient with grade 3 diarrhea and then treatment resumed and completed once diarrhea improved to grade 2. This patient’s diarrhea was considered by the treating physicians to be attributable to his systemic therapy and unlikely related to A-RT. No acute grade 4 or 5 events were observed. There were no late toxicities.

**Table 3:** Acute and late toxicities.

	Patients, no (% of total cohort), n=30					
	Grade 1	Grade 2	Grade 3	Grade 4	Grade 5	Total
Acute*	8 (26.7)	5 (16.7)	**0 (0.0)**	**0 (0.0)**	**0 (0.0)**	13 (43.3)
Abdominal pain	3 (10.0)					3 (10.0)
Nausea	1 (3.3)	2 (6.7)				3 (10.0)
Vomiting	1 (3.3)					1 (3.3)
Diarrhea	1 (3.3)	1 (3.3)				2 (6.7)
Weight loss		1 (3.3)				1 (3.3)
Fatigue	2 (6.7)					2 (6.7)
Anorexia		1 (3.3)				1 (3.3)
	Patients, no (% of total cohort), n=29^†^					
**Late**^‡^	**0 (0.0)**	**0 (0.0)**	**0 (0.0)**	**0 (0.0)**	**0 (0.0)**	**0 (0.0)**

*Acute toxicity was evaluated per the National Cancer Institute’s Common Terminology Criteria for Adverse Events (CTCAE), version 5.0 reporting guidelines, for events occurring within 90 days of the first fraction of A-RT.

^†^One patient died within 90 days of A-RT so was not considered eligible for late toxicity evaluation.

^‡^Late toxicities were those occurring > 90 days from delivery of the first fraction of A-RT and were defined by the Radiation Therapy oncology Group/European Organization for Research and Treatment in Cancer Late Radiation Morbidity Scoring Schema.^27^.

## 6 Discussion

Studies supporting the importance of dose escalation with hypofractionated A-RT in pancreatic cancer have demonstrated substantial prolongation of survival duration when the primary tumor is controlled [15], [28]. Despite evidence demonstrating the benefit of A-RT delivered with conventional linacs using the approach that we have described previously [15], [16], there has not been widespread adoption of this strategy. This lack of implementation is likely due to the challenges of learning to address digestive motion. The MR linac has allowed the introduction of a more straightforward approach to accomplish the same goal. This study is the first to present clinical outcomes in patients with LAPC treated with A-RT using the 1.5 T Unity MR Linac. We were able to begin treating patients earlier than other centers by creating an MR safe compression belt and workflow to address respiratory motion and due to our prior clinical experience delivering ablative doses for LAPC. Other helpful solutions, such as remote real time adaptive re-planning and incorporating MIM into the workflow, have also significantly enhanced efficiency and accuracy of contouring. These tools have enabled promising initial local tumor control at one year with minimal toxicity among patients with tumors millimeters from the radiosensitive GI tract.

Although our study is the first to present results treating LAPC using the Elekta Unity, others have reported results from MR adaptive treatment of LAPC [23], [24], [25]. A multi-institutional study evaluated 44 patients with inoperable pancreatic cancer treated with varied fractionation regimens (55 % treated with BED10 > 70), supporting previously published data on improved survival with BED10 > 70 and demonstrating no grade 3 + toxicities in the high dose group [23]. Another recent study included 44 patients with inoperable pancreatic cancer treated to 50 Gy in 5 fractions without elective nodal coverage using daily online adaptation; however, most patients were treated with the MRIdian Cobalt-60 system and only 6 (14 %) were treated with MR linac [25]. They achieved a 2-year local control rate of 59.3 %, with two grade 3 (4.6 %; gastrointestinal ulcers) and three grade 2 toxicities (6.8 %; duodenal perforation, antral ulcer, gastric bleed). Recently, the Miami Cancer Institute was the first to report encouraging early results after ablative 5-fraction A-RT delivered exclusively on an MR linac [24], yet with lower field strength compared to our system (0.35 T vs 1.5 T). At a median of 10.3 months, they demonstrate a 1-year local control of 87.8 %, similar to our 1-year rate of 78.8 %. Longer follow-up is needed to fully assess long-term local control rates.

Compared to prior studies investigating A-RT for pancreatic cancer, our patient cohort was a higher risk group. Specifically, 13 % of our patients had evidence of metastatic disease prior to A-RT (vs 0–10 % in prior reports) [24], [29], yet our cohort still achieved an excellent 1-year OS compared to modern A-RT studies (80.0 % vs 40 %-74 %) [24], [29]. Consistent with prior literature, disease progression in our cohort was mostly distant (1-year cumulative incidence of 47.4 %). One local disease progression was preceded by a distant metastasis 8 months prior and two patients with local disease progression had synchronous distant metastases. Notwithstanding distant metastases and a higher risk population at baseline, our cohort had an excellent median OS, suggesting that continued efforts to improve systemic therapies after local control is achieved could lead to further improved OS [3]. Treating high risk patients, including challenging cases with tumors directly abutting the GI tract, may explain the patterns of local failure seen in our cohort, as two were marginal failures at the interface of the GTV and a critical, radiosensitive organ. These patterns demonstrate the reality of the trade-off between OAR sparing and ablative dose delivery.

The MR linac planning process was based on our previously described approach to target coverage [16], using planning risk volumes (PRVs) to spare immediately adjacent luminal OARs; however, we used 1 mm rather than 3 mm PRVs. In doing so, OAR constraints were prioritized over GTV coverage along direct tumor-OAR interfaces. This approach was used for safety and results in a heterogeneous dose distribution previously shown to preserve excellent local tumor control despite often incomplete GTV coverage with prescription dose [15]. We previously reported an 8 % rate of grade 3 upper gastrointestional bleeding associated with hypofractionated ablative RT on a conventional linac with CBCT-guidance [15]. In the current study, patients with direct tumor abutment of the duodenum, jejunum, or stomach were treated using the same method and there were no toxicities associated with A-RT. In contrast, severe toxicity after 45 Gy in 5 fractions was reported in a recent phase I dose-escalation study: one grade 4 and one grade 5 gastrointestinal bleed 4.5 and 3 months after SBRT, respectively [29], In a phase II trial of FOLFIRINOX followed by RT to 40 Gy in 5 fractions, four patients (10 %) developed grade 3 or higher toxicities, two of which were grade 5 gastrointestinal bleeding [30]. These severe toxicities emphasize the role that adaptive on-treatment re-planning plays to correct for interfraction and real-time digestive motion. These events may also have been related to the use of planning objectives for the GTV without specification of luminal OAR constraints (e.g., defined as 1 cc under prescription dose). In contrast, our constraints are standardized to the OAR and PRV to control the dose at the interface between the OARs and the GTV.

There is significant opportunity to improve pancreatic cancer-specific image quality on the MR linac. Alternate workflows have been proposed on the 1.5 T MR linac system to either expedite contouring by performing tasks in parallel or implementing research sequences (e.g., 4DMRI, motion averaged) [31]. Our prior study showed that defining the pancreas-duodenum boundary based on a single 3D T2 sequence is often very challenging, thus additional sequences (e.g, T1w contrast, 2D T2w contrast sequence) may improve OAR delineation. In addition, such workflows allowed assessment of potential intrafraction motion when contouring, rather than at the verification or beam-on stage. Our study here is the first to report results of patients with LAPC treated using diagnostic quality (1.5 T field strength) MR adaptive ablative radiation. The outcomes are consistent with prior studies using hypofractionation and CT image guidance [16] as well as the early reports of A-RT delivered with 0.35 T MRgRT [24]. The high field magnet allows for improved soft tissue discrimination and may offer an opportunity to use multiparametric imaging to personalize the radiation dose. Future innovations, such as further accuracy in 3D tracking and autosegmentation, will allow for determination of dose accumulation during motion of the gastrointestinal tract.

This work has uncertainties inherent to retrospective studies. Selection for patients to undergo A-RT on the MR linac over our more fractionated approach may have been biased given these patients often had an anatomically favorable relationship of the tumor to luminal organs. Despite our best efforts, there is potential for incomplete data in the follow-up period that may have limited determination of local tumor progression. For this reason, death was accounted for as a competing risk in the cumulative incidence analysis. Direct comparisons of target coverage from this study to prior reports are limited by variations in dose levels and planning volume margins. The cohort is heterogeneous, including those with small volume metastatic disease who had responded to chemotherapy, limiting direct comparison of results to previously published data from exclusively LAPC populations.

This study is the first to present clinical outcomes of A-RT for LAPC using the 1.5 T Unity MR linac. A prescription dose of 50 Gy in 5 fractions led to excellent 1-year local control with no severe toxicity despite radiosensitive organs adjacent to the target volumes in patients with LAPC. Longer follow-up is needed to fully assess long-term local control rates.

## Declaration of Competing Interest

The authors declare the following financial interests/personal relationships which may be considered as potential competing interests: K.R.T. reports grant funding from the Radiological Society of North America for an unrelated research study. N.T. discloses provision of services from Elekta, Philips (uncompensated), and Sunnybrook Health Sciences Centre. M.R. is a member of the National Comprehensive Cancer Network Panel on Pancreatic Cancer. P.B.R. reports prior research funding from EMD Serono and has received travel support from Elekta. P.B.R. is supported in part by an NIH/NCI grant (K08CA255574) and an NIH Loan Repayment Program (LRP) award. E.M.O reports provision of services from the American Association for Cancer Research; BioNTech; CytomX Therapeutics; HMP Oncology Learning Network; Imedex, Inc.; Integrity Continuing Education, Inc.; Merck & Co Inc.; National Comprehensive Cancer Network; Paradigm Medical Communications, LLC; Physician’s Education Resource; Polaris Group; Rafael Pharmaceuticals, Inc.; Research to Practice; Shanghai Jo’Ann Medical Technology Co., Ltd; Swedish Orphan Biovitrum; WebMD; and twoXar, Inc. A.M.V. reports provision of services from Bristol-Meyers Squibb, GlaxoSmithKline, Lily Oncology, and Silenseed Ltd. D.N.K. reports intellectual property rights with Merck Sharp & Dohme and PsiOxus Therapeutics Ltd. K.Y. reports provision of services from Ipsen Pharma. C.H.C. reports provision of services from AstraZeneca, Elekta, and Trisalus. C.H.C. has ownership/equity interests in Oncternal Therapeutics, Inc.
