# Supplementary material for: Stereotactic ablative radiation for pancreatic cancer on a 1.5 Telsa magnetic resonance-linac system
Source: Phys Imaging Radiat Oncol. 2022 Oct 28;24:88–94. doi: 10.1016/j.phro.2022.10.003 (PMC9640311; doi:10.1016/j.phro.2022.10.003)
Supplement: Supplementary data 1 [file mmc1.docx]

**SUPPLEMENTARY MATERIAL**

**
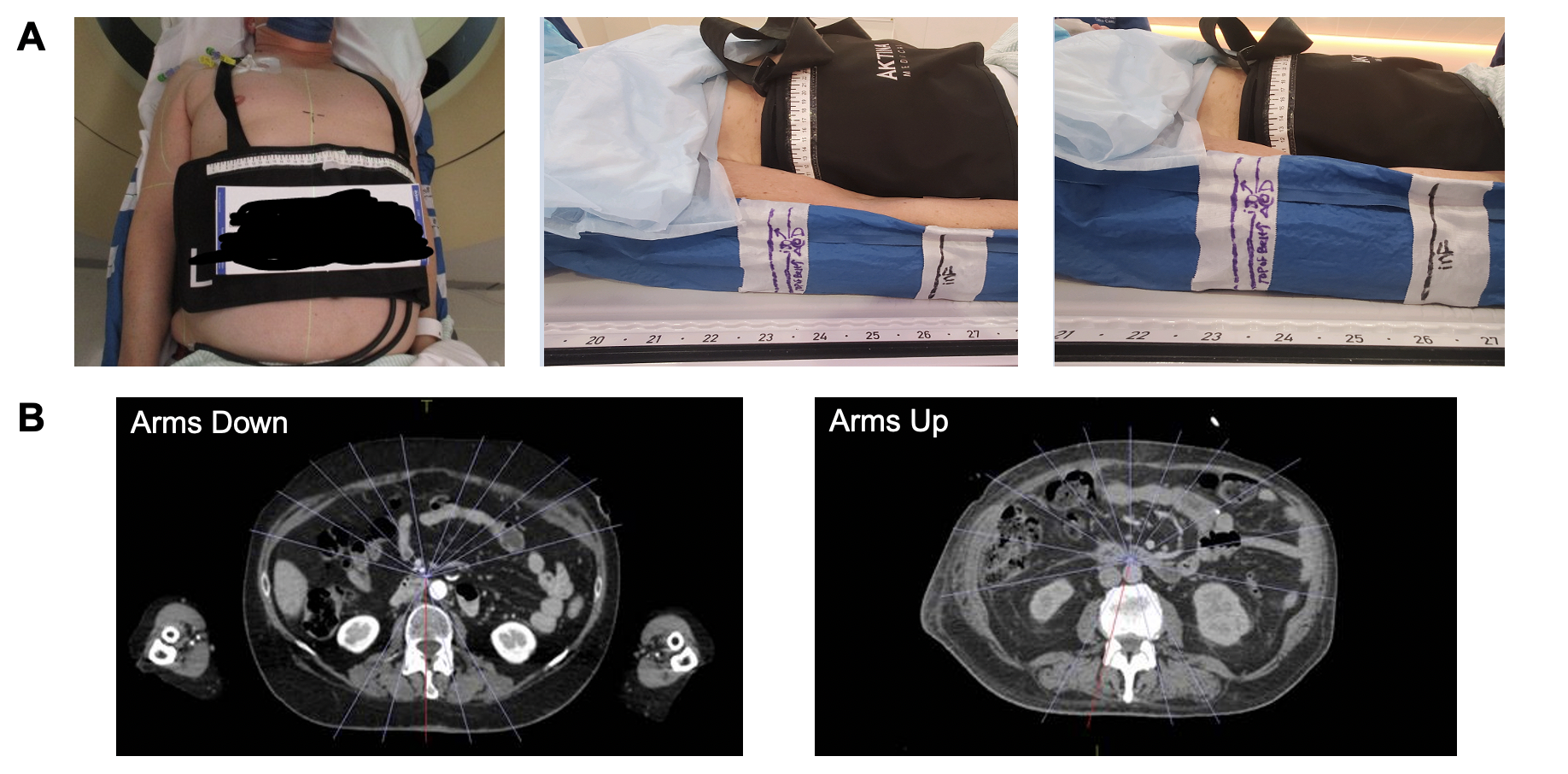
**

**Supplementary Figure 1. Patient set-up with immobilization and beam arrangement**

A) Patient set-up using abdominal compression for motion management on the Elekta Unity system. The patients are simulated with arms down and posteriorly (as opposed to arms up) for improved tolerance. Patients are immobilized in a Q-fix device and custom Vac-lok that wraps around the arms as much as possible. Tattoos were placed just above the arms to help with the setup. The belt (Aktina Medical, Congers, NY) is a corset and has been modified in-house to ensure MR safety and compatibility. An air bladder under the belt is connected to a sphygmomanometer that ensures reproducible pressure is applied during simulation as well as treatment. The pressure is applied to minimize the gross tumor volume (GTV) and nearby OAR motion to be within 5 mm and monitored and controlled from the console area. Patients underwent both CT and MR simulation. B) Beam arrangement in ‘arms down’ position (left) and ‘arms up’ position (right). Fifteen circumferential beams excluding gantry angles through a specific high density couch structure, arms and through the cryostat pipe were used for generating a reference plan on CT using Monaco Monte Carlo based dose calculation system.
