# Supplementary material for: Stereotactic ablative radiation for pancreatic cancer on a 1.5 Telsa magnetic resonance-linac system
Source: Phys Imaging Radiat Oncol. 2022 Oct 28;24:88–94. doi: 10.1016/j.phro.2022.10.003 (PMC9640311; doi:10.1016/j.phro.2022.10.003)
Supplement: Supplementary data 2 [file mmc2.docx]

**SUPPLEMENTARY MATERIAL**


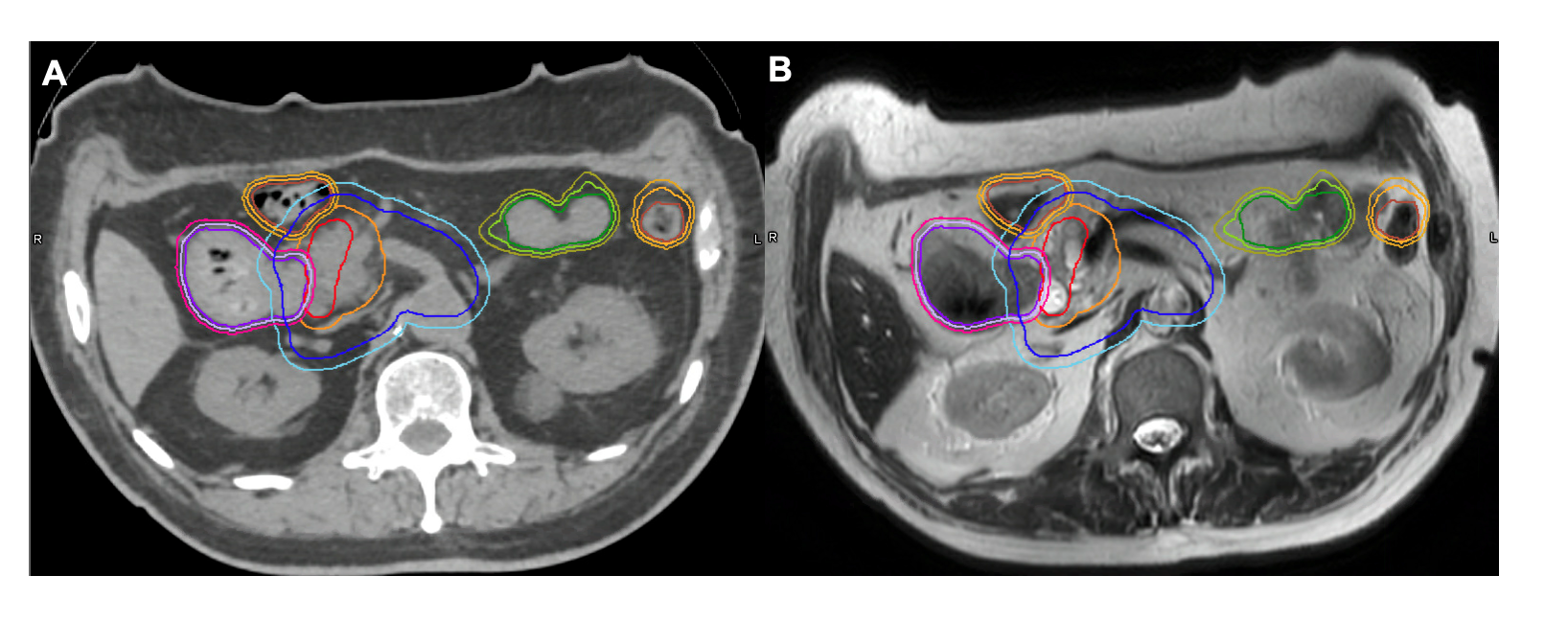


**Supplementary Figure 2**

Target and organ-at-risk (OAR) contours and planning volumes shown on CT (A) and T2 weighted MRI (B). The gross tumor (GTV) target dose was 50Gy in 5 fractions (BED assuming ⍺/𝛽=10, BED10=100Gy) along with a second dose level of 25 or 33 Gy in 5 fractions to the volume at risk of harboring microscopic disease in all patients. Elective nodal coverage included peripancreatic nodes within 1.5 cm of the gross tumor and celiac and mesenteric artery nodes. Out of necessity, the gross tumor volume (GTV) coverage was heterogeneous in most cases, particularly along the GTV-OAR interface. A gradient from the GTV prescription dose to the OAR tolerance dose is expected at these interfaces, and incomplete coverage of GTV with prescription dose is expected with a goal of 80% coverage with the ablative dose. Three GI OARs were contoured: stomach with the first two segments of the duodenum (purple), the remaining small bowel (green), and the large bowel (brown). PTV50 (orange) was created using a 5 mm margin to the GTV (red). A 3 mm expansion was added to all OARs and excluded from the planning target volume (PTV) prescribed to receive 50 Gy (PTV50). A 1 mm planning risk margin was added to the OARs. The microscopic PTV (25-33 Gy) was created using a 15 mm margin from GTV as well as celiac axis and the superior mesenteric artery nodal basins (CTV25 and PTV25 shown here in blue).
