# Supplementary material for: Stereotactic ablative radiation for pancreatic cancer on a 1.5 Telsa magnetic resonance-linac system
Source: Phys Imaging Radiat Oncol. 2022 Oct 28;24:88–94. doi: 10.1016/j.phro.2022.10.003 (PMC9640311; doi:10.1016/j.phro.2022.10.003)
Supplement: Supplementary data 3 [file mmc3.docx]

**SUPPLEMENTARY MATERIAL**


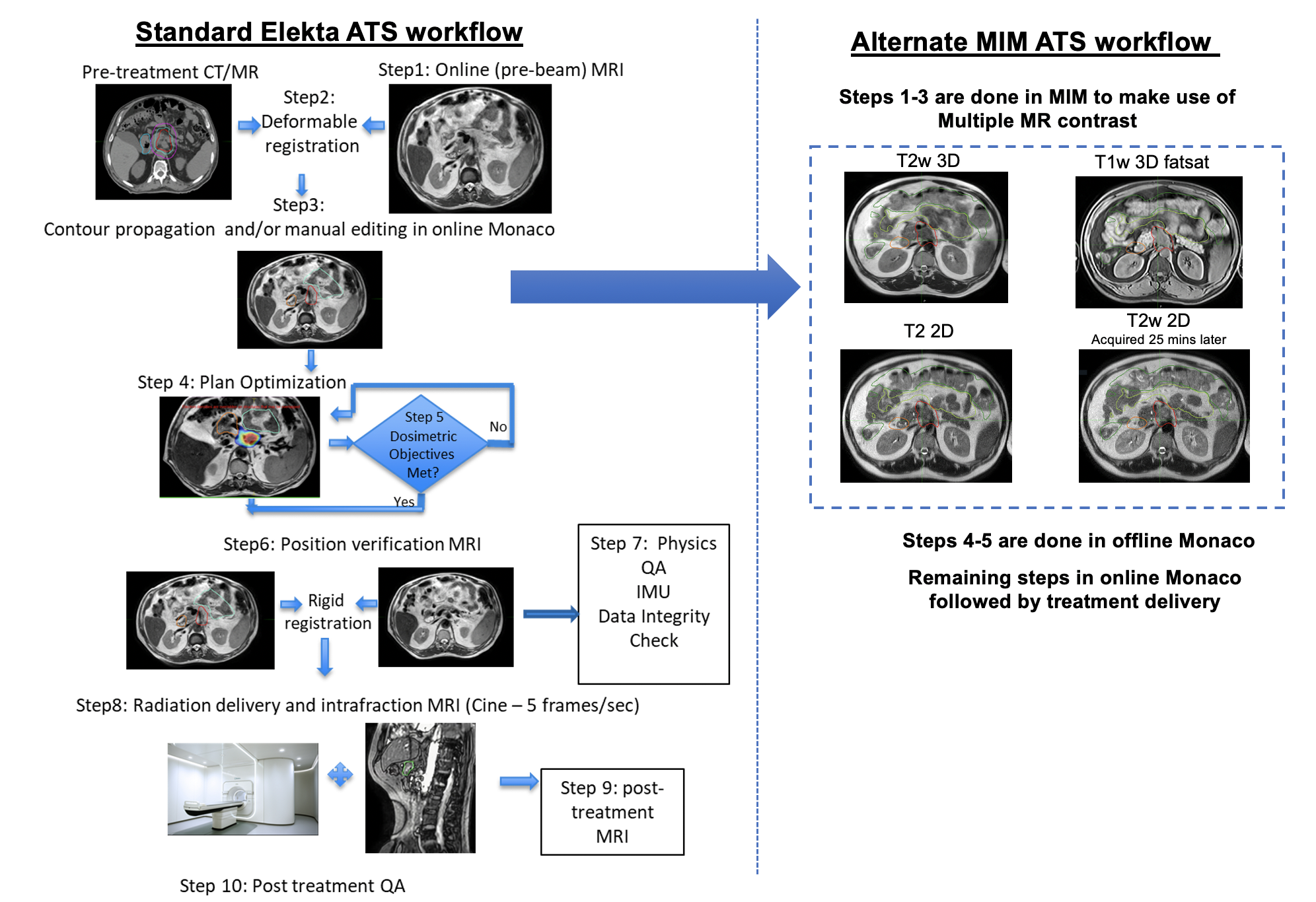


**Supplementary Figure 3.** **Standard Elekta Adapt-to-Shape (ATS) workflow and its modification to enable contouring in MIM Vista for use of multiple MR sequences.**

Each patient underwent 5 fraction ablative radiation treatment with daily online plan adaptation using Elekta’s ATS workflow, which was modified to enable contouring in MIM VISTA that allowed the use of multiple MR sequences (instead of a single 3D sequence) for contouring GTV and OARs. Alternate MIM workflow for online adaptive planning also enabled physicians to perform contouring remotely during the COVID-19 pandemic since MIM could be accessed via CITRIX application. Our previously published experience with this workflow reported a median treatment time-per-fraction from patient setup to room exit of 75 minutes.
