# Supplementary material for: Stereotactic ablative radiation for pancreatic cancer on a 1.5 Telsa magnetic resonance-linac system
Source: Phys Imaging Radiat Oncol. 2022 Oct 28;24:88–94. doi: 10.1016/j.phro.2022.10.003 (PMC9640311; doi:10.1016/j.phro.2022.10.003)
Supplement: Supplementary data 4 [file mmc4.docx]

**SUPPLEMENTARY MATERIAL**


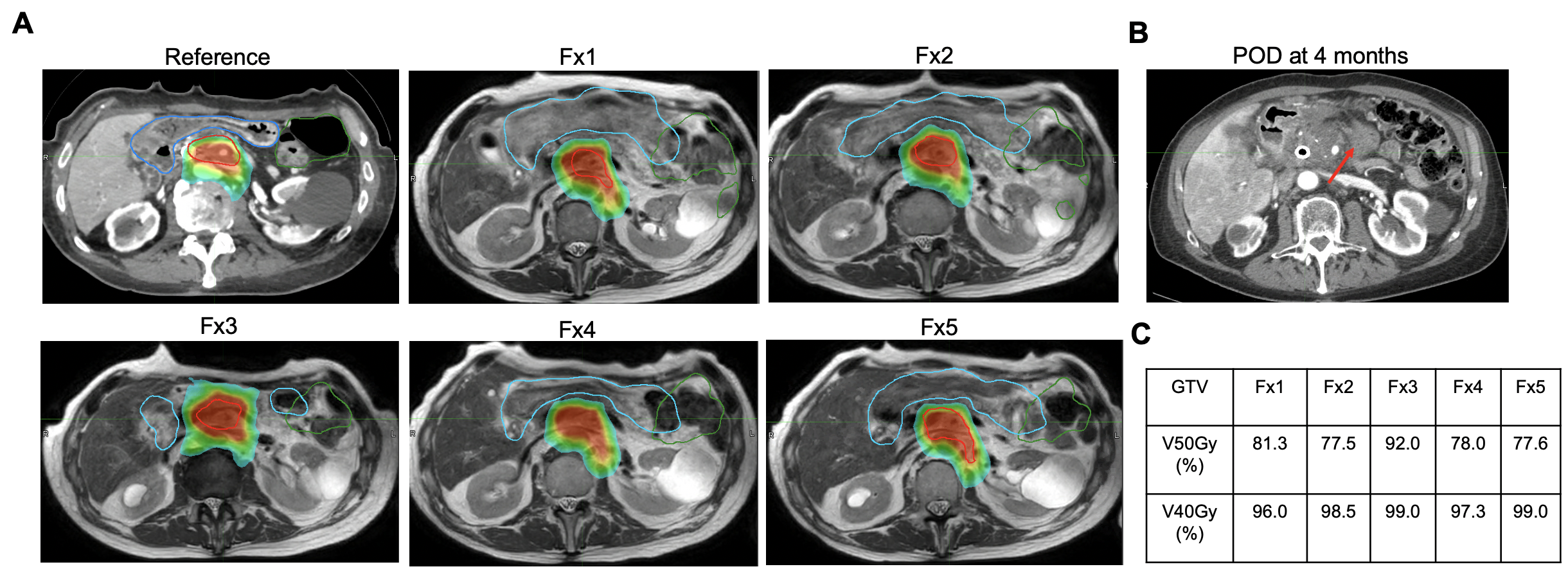


**Supplementary Figure 4. Dosimetry for a case of local progression**

A) Dose color-wash pre-treatment and for each fraction (fx 1-5) with stomach (blue) wrapping around the GTV (red). B) CT at 4 months post-ablative RT demonstrates progression of disease (POD). C) GTV dosimetry for each fraction summarized in a table.

Abbreviations: Fx, fraction; GTV, gross tumor volume
